# Supplementary material for: NFATc3 Enhances DREAM Complex-Driven Transactivation
Source: bioRxiv. 2026 Jan 12:2026.01.12.699082. Preprint. [Version 1] doi: 10.64898/2026.01.12.699082 (PMC12871376; doi:10.64898/2026.01.12.699082)

bioRxiv preprint doi: https://doi.org/10.64898/2026.01.12.699082; this version posted January 12, 2026. The copyright holder for this preprint (which was not certified by peer review) is the author/funder. This article is a US Government work. It is not subject to copyright under 17 USC 105 and is also made available for use under a CC0 license.

Supplementary Information

Supplementary Figure 1. Physical interaction of NFATc3 with the MuvB complex and FOXM1. A. Ectopic expression and co-immunoprecipitation (coIP) in H1792 cells. Cells were transiently transfected with empty vector, control FLAG, or FLAG-tagged NFATc3. Cell lysates were immunoprecipitated with an anti-FLAG, followed by immunoblotting (IB) with antibodies against FOXM1, LIN9, and LIN54. **B.** Endogenous co-immunoprecipitation (coIP) in H1792 cells using IgG (control) or anti-NFATc3. Non-specific IgG served as a negative control.

bioRxiv preprint doi: https://doi.org/10.64898/2026.01.12.699082; this version posted January 12, 2026. The copyright holder for this preprint (which was not certified by peer review) is the author/funder. This article is a US Government work. It is not subject to copyright under 17 USC 105 and is also made available for use under a CC0 license.

Supplementary Figure 1

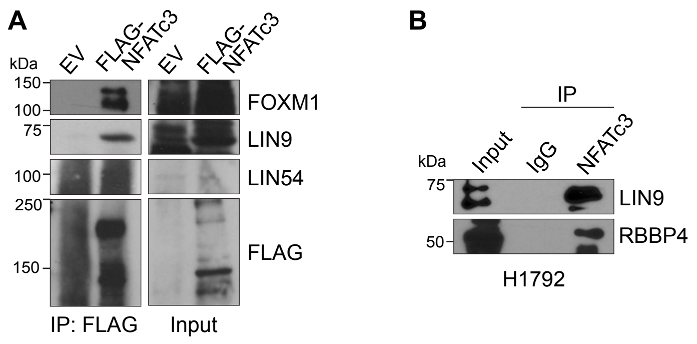

Supplement: Supplement 1 [file NIHPP2026.01.12.699082v1-supplement-1.pdf]
